# A New Criterion for the Application of 2-Stage Implant-Only Breast Reconstruction Using a Classification Based on the Rostrocaudal Distance Along the Chest Wall Between the Lowest Point of the Breast and Inframammary Fold

**Published:** 2017-08-18

**Authors:** Naohiro Ishii, Jiro Ando, Michiko Harao, Masaru Takemae, Kazuo Kishi

**Affiliations:** ^a^Department of Plastic and Reconstructive Surgery, Keio University, Shinjyuku Ward, Tokyo, Japan; ^b^Department of Breast Surgery, Tochigi Cancer Center, Utsunomiya City, Tochigi, Japan

**Keywords:** breast reconstruction, breast implant, inframammary fold, breast ptosis, tissue expander

## Abstract

**Objective:** It is generally difficult to achieve symmetry in implant-only breast reconstruction with ptosis, and it remains unclear what quantitative criterion may be applied in cases of breast ptosis regarding the application of this modality. Our study aimed to suggest a criterion for obtaining good aesthetic outcomes and a symmetrical inframammary fold that is well-fitting for the brassiere in implant-only breast reconstruction after simple mastectomy. **Methods:** We classified into 3 groups 50 consecutive patients who underwent implant-only breast reconstruction that created an inframammary fold using a modified internal method after simple mastectomy. The classification was based on the rostrocaudal distance along the chest wall between the lowest point of the breast the and the inframammary fold on the contralateral side (Dc, in millimeters). Thereafter, we compared this distance on the reconstructed side (Dr, in millimeters), Dc−Dr, and projection of the implant (Pi, in centimeters) between the groups and investigated the correlation between Dr and lower (Pi < 5.0 cm) and higher Pi (Pi ≥ 5.0 cm). **Results:** Dr was similar to Dc in groups 1 and 2 (0 ≤ Dc < 10 mm, 40/50 [80%]); however, Dr was significantly lesser than Dc in group 3 (Dc ≥ 10 mm, 10/50 [20%]). In addition, we found significant positive correlations between Dr and lower Pi and between Dr and higher Pi. **Conclusions:** A Dc below 10 mm may be a good indication for implant-only breast reconstruction. Furthermore, the modality may be applied where Dc is 7 mm or less in cases with lower Pi and where Dc is 13 mm or less in cases with higher Pi.

Implant-based breast reconstruction techniques are widely used because they are less invasive. To achieve good aesthetic outcomes, the shape and position of the reconstructed breast should be symmetrical. In addition, considering a proper fitting for the brassiere, the inframammary fold (IMF) should be as symmetrical as possible. However, in implant-only breast reconstruction with ptosis, it is generally difficult to achieve such symmetry.

To achieve symmetry of both breasts in severe ptosis, revision surgery of the contralateral breast or reconstruction with autologous tissue is needed. Therefore, it is important to evaluate the degree of ptosis before deciding on the method of breast reconstruction. Regnault's^1^ classification was popularly used to evaluate the degree of ptosis; however, it seemed to be established considering the relationship between the position of the nipple and the lower breast pole and is therefore not suitable for implant-only breast reconstruction after simple mastectomy. To date, no study has provided any quantitative criteria that clarify the cases of breast ptosis that may undergo this procedure.

Our study aimed to evaluate degree of breast ptosis based on the rostrocaudal distance between the IMF to the lowest point of the breast and suggest a useful and practical criterion for obtaining good aesthetic good outcomes and a symmetrical IMF for satisfactory brassiere fitting in the reconstructed breast with respect to implant-only breast reconstruction after simple mastectomy. Furthermore, it seemed necessary not only to predict the need for a contralateral side operation preoperatively but also to suggest conversion to autologous reconstruction when unsatisfactory outcomes are expected.

## MATERIALS AND METHODS

### Patients

We prospectively investigated 50 consecutive patients who underwent unilateral, 2-stage, implant-only breast reconstruction after simple mastectomy between April 2014 and August 2016. We used an anatomical and textured silicone breast implant (Natrelle 410, True form 3; Allergan, Ireland, Dublin) for the procedures. Patients who received radiation therapy were excluded. The following characteristics were noted in the patient cohort: age, range = 18 to 71 (mean 48) years; body mass index, range = 15.5 to 27.3 (mean 21.3) kg/m^2^; period of measurement, range = 6 to 12 (mean 9.5) months postoperatively; follow-up period, range = 8 to 36 (mean 20.9) months postoperatively; and breast implant weight, range = 90 to 620 (mean 342.7) g.

### Surgical procedure

In the first stage, a tissue expander ([TE] Natrelle 133; Allergan, Ireland, Dublin) was covered completely with well-vascularized tissue composed of the pectoralis major muscle, serratus anterior fascia (occasionally including a portion of the serratus anterior muscle), and rectus abdominis fascia, using the musculofascial pocket method. For expanding the skin at the lower pole mainly, the TE was placed at the lower border, 2 fingers below the IMF at the contralateral side. We performed an overexpansion with the TE, particularly in cases of breast ptosis. The extent of tissue expansion was determined by the quality of skin and weight of the breast implant. The rate of expansion (weight of TE with full saline/weight of the breast implant) ranged from 1.1 to 2.4 (mean = 1.4).

In the second stage (usually 5-8 months after TE placement), the TE was replaced with the breast implant. Breast implant insertion was performed using previous incisions. After cutting the capsule and removing caudal soft tissue, including the superficial fascia, the IMF was created using a modified internal method by Nava et al^2^ and Bogetti et al.^3^ Instead of No. 0 absorbable polyfilament sutures, we used 3-0 absorbable 6 to 7 horizontal mattress sutures (Vicryl; Ethicon, Somerville, NJ).

For 6 months after breast implant insertion, patients fixed both breasts with a controlling brassiere. All reconstruction procedures and measurements were performed by the first author.

### Measurement and classification

**Figure 1:**
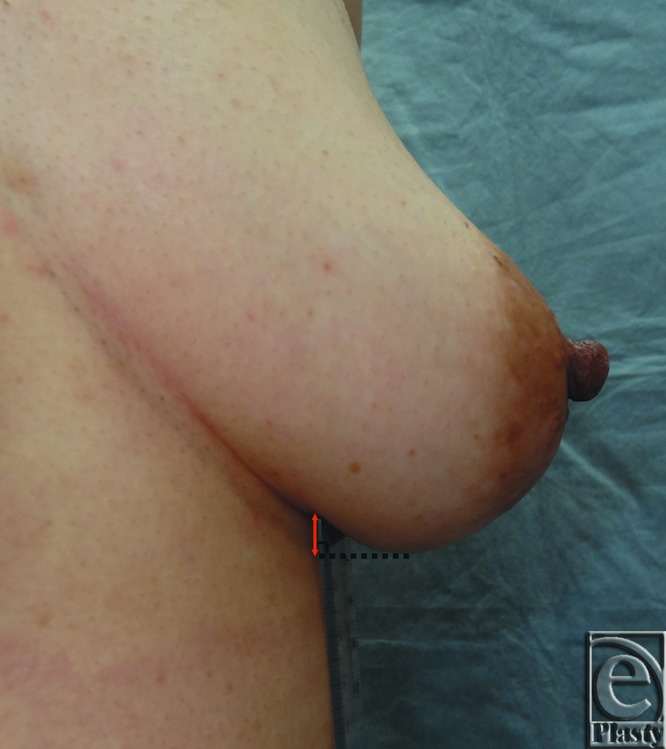
We measured the rostrocaudal distance along the chest wall in the anterior view between the lowest point of the breast and the IMF on the reconstructed and contralateral sides. A red 2-headed arrow shows the measurement parallel to the body axis between the point on the chest wall vertical to the lowest point of the breast and the point on the IMF. IMF indicates inframammary fold.

**Figure 2:**
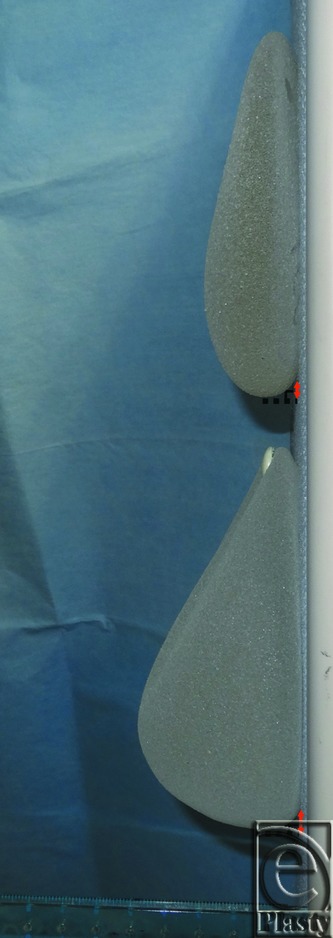
Silicone breast implants, sagittal view. The superior implant has a low projection (2.9 cm), and the inferior implant has a high projection (5.5 cm). The distance along the wall in the anterior view between the point on the wall vertical to the lowest point of the implant and the point created as the inframammary fold in the implant is 3 mm in the implant above and 12 mm in the implant below.

We measured the rostrocaudal distance along the chest wall in the anterior view between the lowest point of the breast and the IMF on the reconstructed side (Dr) and also measured this distance on the contralateral side (Dc), after more than 6 months post–breast implant insertion (Fig 1). If the lowest point of the breast was higher than the IMF, we defined each value as zero. The patient population was classified into 3 groups based on Dc, Dr, and Dc−Dr, and the projection of the breast implant (Pi) was compared between the groups. In each group, boundary values were defined as 0 mm (no ptosis/minimal to moderate ptosis) and 10 mm (minimal to moderate ptosis/moderate to marked ptosis). Considering the shape of the breast implant, the difference in Dr between the lower and higher Pi groups could be found (Fig 2); therefore, we investigated the correlation between Dr and lower Pi and between Dr and higher Pi.

### Statistics

Data were analyzed using the Statistical Package for the Social Sciences for Windows, version 23 (IBM Corp, Chicago, Ill). Holm's test was used to compare the means of continuous variables (Dr, Dc−Dr, and Pi) between the 3 groups. Simple linear regression was used to define the linear relationship between Dr and lower Pi and between Dr and higher Pi. Student's *t* test was used to compare continuous variables between Dr in lower Pi and Dr in higher Pi. For all statistical tests, a *P* value less than .05 was considered statistically significant.

### Ethics

The protocol for this study was approved by the institutional review board. All patients provided written informed consent before publication of this article. Our study was conducted in accordance with the Declaration of Helsinki.

## RESULTS

**Figure 3:**
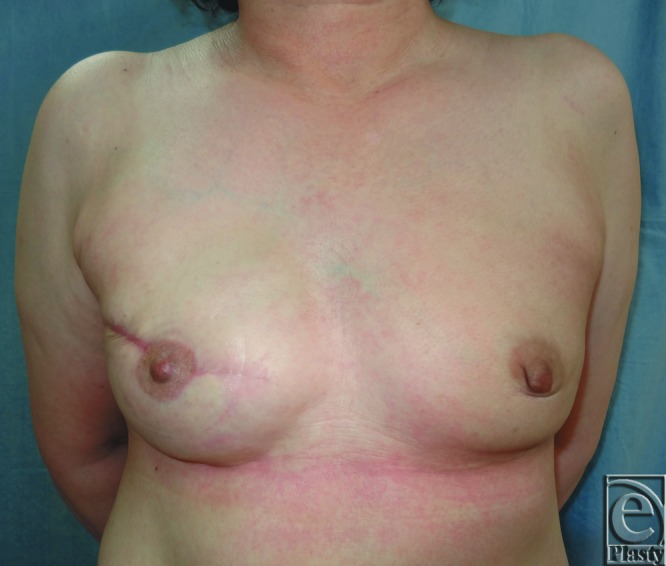

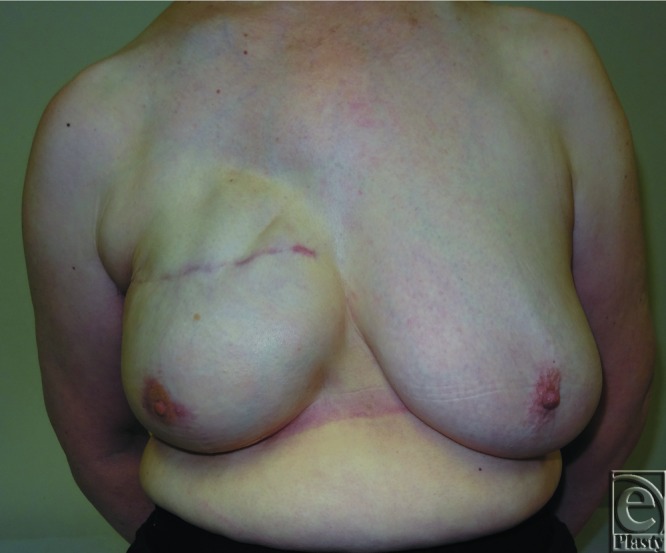
(a) Postoperative photograph of a group 1 patient (reconstructed side; left, no ptosis). Dc = 0 mm, Dr = 0 mm, Pi = 3.4 cm. (b) Postoperative photograph of a group 2 patient (reconstructed side; right, low-to-moderate ptosis). Dc = 7 mm, Dr = 7 mm, Pi = 6.1 cm. Dc and Dr indicate the rostrocaudal distance along the chest wall in the anterior view between the lowest point of the breast and the inframammary fold on the contralateral (Dc) and reconstructed sides (Dr); Pi, the projection of the breast implant.

None of the patients experienced TE or breast implant failure due to infection or extrusion, necrosis of the skin envelope, and definite postoperative capsule contracture (Baker's classification,^4^ ≥3). In all patients, Dc ranged from 0 to 20 (mean = 4.6) mm; Dr, 0 to 15 (mean = 3.8) mm; Dc−Dr, −4 to 15 (mean = 0.8) mm; and Pi, 2.0 to 7.0 (mean = 5.0) cm. The characteristics of the patients, according to group, are shown in Table 1. There were 16 (32%) group 1 (no ptosis), 24 (48%) group 2 (minimal to moderate ptosis), and 10 (20%) group 3 (moderate to marked ptosis) patients. Two typical cases are shown in Figure 3. Two patients (Dc−Dr: 13 and 15 mm) in group 3 complained that the brassiere did not fit well. One of them underwent revision surgery to change the position of the breast implant and improve the fitting.

**Figure 4:**
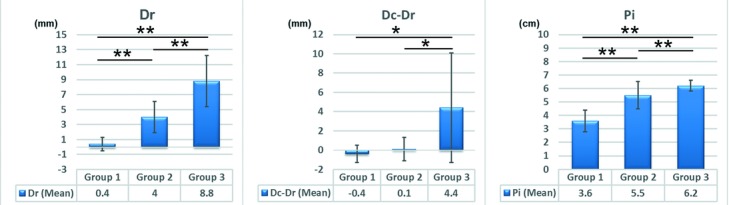
Bar chart showing a comparison of Dr, Dc−Dr, and Pi according to group. Error bars represent the standard deviation. *P < .05, **P < .01. Dc and Dr indicate the rostrocaudal distance along the chest wall in the anterior view between the lowest point of the breast and the inframammary fold on the contralateral (Dc) and reconstructed sides (Dr); Pi, the projection of the breast implant.

Dr was significantly longer in group 2 than in group 1, in group 3 than in group 1, and in group 3 than in group 2 (*P* < .01, all). Dc−Dr was significantly longer in group 3 than in group 1 and in group 3 than in group 2 (*P* < .05, both). No significant difference was noted in Dc−Dr between group 1 and group 2 (*P* = .18). Pi was significantly longer in group 2 than in group 1, in group 3 than in group 1, and in group 3 than in group 2 (*P* < .01, all) (Fig 4).

**Figure 5:**
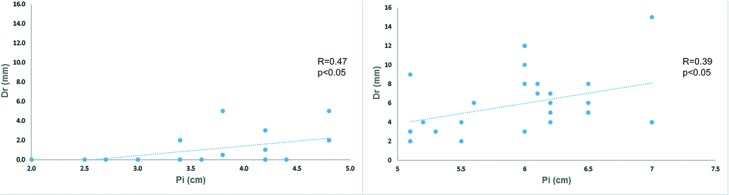
Scatter plots with simple linear regression of Dr and lower Pi (Pi <5.0 cm) and Dr and higher Pi (Pi ≥5.0 cm). Dr indicates the rostrocaudal distance along the chest wall in the anterior view between the lowest point of the breast and the inframammary fold on the reconstructed side; Pi, the projection of the breast implant.

Dr tended to be longer when Pi was greater than 5.0 cm; we defined the boundary values between the lower and higher Pi groups as 5.0 cm. Significant positive correlations were noted between Dr and lower Pi (*R* = 0.47, *P* < .05) and between Dr and higher Pi (*R* = 0.39, *P* < .05) (Fig 5). The difference between Dr in the lower Pi group and Dr in the higher Pi group was significant and considering the mean ± 95% confidence interval in Dr, Dr in the lower Pi group can attain a length of 1.8 mm and Dr in the higher Pi group can attain a length of 7.2 mm (Table 2).

## DISCUSSION

This prospective study aimed to investigate the classification of patients who underwent unilateral, 2-stage, implant-only breast reconstruction using a modified internal method by Nava et al^2^ and Bogetti et al^3^ after simple mastectomy. The patients were classified on the basis of Dc into 3 groups, and Dr, Dc−Dr, and Pi were compared between the groups. We found a positive relationship between Dc and Dr and Pi and that Dr was similar to Dc in groups 1 and 2; however, Dr was significantly shorter than Dc in group 3. On the basis of this study, classification into group 1 or 2 (0 ≤ Dc < 10 mm) may be a good indication for implant-only breast reconstruction. In addition, we found significant positive correlations between Dr and lower Pi and between Dr and higher Pi.

In breast reconstruction with ptosis, it is necessary to achieve a symmetrical IMF with sufficient depth and acceptable aesthetics in order to achieve a good shape and position, as well as a comfortable fit for the brassiere. The skin envelope decreases postoperatively compared with preoperatively after simple mastectomy. Furthermore, the shape of the breast implant is different from the shape of the ptotic breast in the sagittal plane (Fig 2); therefore, an elaborate technique is needed to create a symmetrical IMF with sufficient depth and acceptable aesthetics as described earlier. At first, a TE should be inserted to expand the lower pole mainly and overexpansion should be performed.^5^^,^^6^ Various methods for creating a satisfactory IMF, both external and internal, have been reported.^2^^,^^3^^,^^6^^-^9 We used a modified internal method by Nava et al and Bogetti et al because of its ability to achieve a satisfactory and less retrogressive IMF without additional scarring.

Larger implant volume was a significant predictor of better IMF outcomes according to photogenic assessment, and increased breast projection with implants has some corrective effects on ptosis of the reconstructed breast.^5^^,^^10^ However, compared with breast reconstruction using autologous tissue, it is often difficult to create a symmetrical IMF with sufficient depth and aesthetic outcomes in implant-only breast reconstruction with ptosis.^9^ On the basis of clinical experience, it is difficult to create such an IMF if breast ptosis is marked; however, although the criteria for application using a numerical value have been sought, to the best of our knowledge, no such criteria have been previously suggested.

The gap between Dc and Dr may be acceptable until a length of 5 to 6 mm considering the allowance of minimal asymmetry for brassiere fitting and therefore the application of implant-only breast reconstruction should be for cases where Dc is 7 mm or less (group 1, all; group 2; partial) for the lower Pi group (Pi <5.0 cm) and where Dc is 13 mm or less (groups 1 and 2, all; group 3, partial) for the higher Pi group (Pi ≥ 5.0 cm).

The skin envelope tends to constrict according to the shape of the breast implant. Therefore, even if overexpansion of the skin envelope is performed and an IMF is created with firm fixation, it is difficult to achieve a symmetrical IMF in implant-only breast reconstruction with moderate ptosis using a breast implant with a low projection, because the difference in the application of the procedure between cases in the lower and higher Pi groups becomes apparent.

The rostrocaudal distance along the chest wall between the lowest point of the breast and the IMF is a useful criterion for the application of implant-only breast reconstruction after simple mastectomy. Furthermore, patients may be informed, with better evidence than before, of the need to reshape the contralateral breast and the desirability of reconstruction using autologous tissue to achieve symmetry of both breasts.

Our study could have included a control group using other techniques of IMF creation, other mastectomy methods (skin-sparing mastectomy, nipple-sparing mastectomy), different silicone breast implant types (round, smooth, other consistencies), and/or receiving radiotherapy. However, breast reconstruction after simple mastectomy using the anatomical silicone breast implant is the most popular modality; thus, our study seems relevant.

## CONCLUSION

We classified patients who underwent implant-only breast reconstruction after simple mastectomy into 3 groups based on Dc and compared the Dr, Dc−Dr, and Pi between these groups. Moreover, we determined the correlation between Dr and lower Pi and between Dr and higher Pi. Classification into group 1 or 2 (0 ≤ Dc <10 mm) may be a good indication for implant-only breast reconstruction. In addition, on the basis of Dc, we showed the application of this reconstruction technique in patients with lower Pi and higher Pi. We believe that Dc with reference to Pi is a simple, practical, and useful criterion for the application of implant-only breast reconstruction after simple mastectomy.

## Figures and Tables

**Table 1:** Participant classification into three groups based on Dc*

Group; cases	Dc, mm	Dc, mean ±SD, mm	Age, mean ± SD, y	Body mass index, mean ± SD, kg/m^2^	Rate of expansion, mean ± SD
1; N = 16	Dc = 0	0.0 ± 0.0	45.6 ± 9.8	19.5 ± 1.8	1.3 ± 0.1
2; N = 24	0 < Dc < 10	4.0 ± 2.2	47.3 ± 10.3	21.9 ± 2.5	1.4 ± 0.2
3; N = 10	10 ≤ Dc	13.2 ± 3.7	53.6 ± 10.4	22.7 ± 1.8	1.5 ± 0.2

*Dc indicates the rostrocaudal distance along the chest wall between the lowest point of the breast and the inframammary fold on the contralateral side; rate of expansion, the weight of the tissue expander with full saline/the weight of the silicone breast implant.

**Table 2:** Dr in the lower and higher Pi groups*

Pi, cm; cases	Dr, mean ± 95% confidence interval, mm
Lower Pi, Pi < 5.0; N = 21	1.1 ± 0.7
Higher Pi, Pi ≥ 5.0; N = 29	5.9 ± 1.3
	*P* < .01

*Dr indicates the rostrocaudal distance along the chest wall between the lowest point of the breast and the inframammary fold on the reconstructed side. Pi, the projection of the silicone breast implant.
